# *N*,*N*,*N*-Trimethyl-*N*-(methyl 5-de­oxy-2,3-*O*-iso­propyl­idene-β-d-ribo­furan­osid-5-yl)ammonium 4-methyl­benzene­sulfonate sesquihydrate

**DOI:** 10.1107/S1600536813014797

**Published:** 2013-06-08

**Authors:** Barbara Dmochowska, Karol Sikora, Jaroslaw Chojnacki, Wieslaw Wojnowski, Andrzej Wiśniewski

**Affiliations:** a Department of Chemistry, University of Gdansk, Sobieskiego 18, PL-80952 Gdańsk, Poland; b Department of Chemistry, University of Gdansk, Sobieskiego 18, Gdańsk, PL-80952, Poland; c Department of Chemistry, Gdańsk University of Technology, G. Narutowicza Str. 11/12, PL-80233 Gdańsk, Poland

## Abstract

The structure of the title compound, [C_12_H_24_NO_4_][C_7_H_7_O_3_S]·1.5H_2_O, contains alternating layers parallel to (001) of hydro­phobic and polar character, stabilized by C—H⋯O hydrogen bonding. The furan ring adopts an envelope conformation with the C(OMe) atom as the flap, and the dioxolane ring is twisted about one of the O—C(methine) bonds. A comparison to related compounds is presented. The tosyl­ate-O atoms were disordered over two positions with the major component having a site occupancy factor = 0.566 (12). The structure was refined as a rotary twin with regard to rotation about the *c* axis with the contribution of the second component being 0.0048 (6). Solvate water mol­ecules are highly disordered and were removed using the SQUEEZE procedure; the unit cell characteristics take into account the presence of the disordered solvent. High-resolution ^1^H and ^13^C NMR spectroscopic data are also presented.

## Related literature

For background to quaternary ammonium compounds, see: Jones (1984 ▶); Śliwa (1996 ▶); Sajomsang *et al.* (2009 ▶); Obłąk & Gamian (2010 ▶); Binks *et al.* (2011 ▶); Singh *et al.* (2009 ▶); Cruz-Guzman *et al.* (2005 ▶); Rabea *et al.* (2003 ▶); Belalia *et al.*, 2008 ▶; McDonnell & Russell (1999 ▶); Boethling (1984 ▶); Levinson (1999 ▶); Cross & Singer (1994 ▶). For QAC sugar derivatives, see: Abel *et al.* (2002 ▶); Blizzard *et al.* (2002 ▶); Honda *et al.* (1988 ▶); Thomas *et al.* (2009 ▶); Maslov *et al.* (2010 ▶); Dmochowska *et al.* (2006 ▶, 2009 ▶, 2011 ▶); Pellowska-Januszek *et al.* (2004 ▶); Skorupa *et al.* (2004 ▶). For related synthetic methods, see: Gosh & Liu (1996 ▶); Sairam *et al.* 2003 ▶; Sarabia-Garcia & Lopez-Herrera (1996 ▶); Dibrov *et al.* (2010 ▶). For ring puckering analysis, see: Cremer & Pople (1975 ▶). 
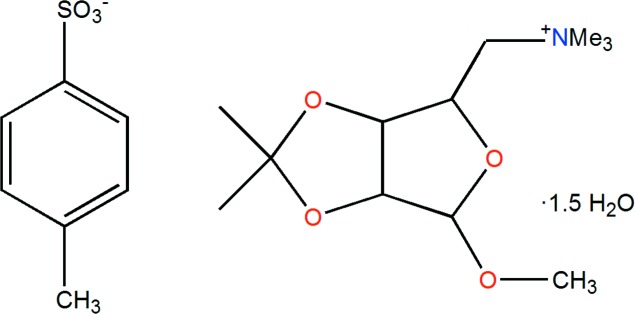


## Experimental

### 

#### Crystal data

C_12_H_24_NO_4_^+^·C_7_H_7_O_3_S^−^·1.5H_2_O
*M* *_r_* = 444.53
Monoclinic, 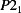

*a* = 11.4896 (15) Å
*b* = 7.9311 (11) Å
*c* = 13.4853 (17) Å
β = 111.619 (12)°
*V* = 1142.4 (3) Å^3^
*Z* = 2
Mo *K*α radiation
μ = 0.19 mm^−1^
*T* = 200 K
0.37 × 0.2 × 0.17 mm

#### Data collection

Kuma KM4CCD (Sapphire2 detector) diffractometer
26053 measured reflections
4481 independent reflections
4325 reflections with *I* > 2σ(*I*)
*R*_int_ = 0.038

#### Refinement

*R*[*F* ^2^ > 2σ(*F* ^2^)] = 0.051
*wR*(*F* ^2^) = 0.139
*S* = 1.07
4481 reflections
283 parameters
40 restraints
H-atom parameters constrained
Δρ_max_ = 0.40 e Å^−3^
Δρ_min_ = −0.31 e Å^−3^
Absolute structure: Flack & Bernardinelli (1999 ▶)
Flack parameter: 0.08 (12)

### 

Data collection: *CrysAlis CCD* (Oxford Diffraction, 2006 ▶); cell refinement: *CrysAlis CCD*; data reduction: *CrysAlis RED* (Oxford Diffraction, 2006 ▶); program(s) used to solve structure: *SHELXS97* (Sheldrick, 2008 ▶); program(s) used to refine structure: *SHELXL97* (Sheldrick, 2008 ▶) and *WinGX* (Farrugia, 2012 ▶); molecular graphics: *Mercury* (Macrae *et al.*, 2006 ▶); software used to prepare material for publication: *PLATON* (Spek, 2009 ▶) and *publCIF* (Westrip, 2010 ▶).

## Supplementary Material

Crystal structure: contains datablock(s) global, I. DOI: 10.1107/S1600536813014797/tk5228sup1.cif

Structure factors: contains datablock(s) I. DOI: 10.1107/S1600536813014797/tk5228Isup2.hkl

Additional supplementary materials:  crystallographic information; 3D view; checkCIF report

## Figures and Tables

**Table 1:** Hydrogen-bond geometry (Å, °)

*D*—H⋯*A*	*D*—H	H⋯*A*	*D*⋯*A*	*D*—H⋯*A*
C13—H13*A*⋯O28*A* ^i^	0.98	2.45	3.412 (15)	167
C13—H13*B*⋯O28*A* ^ii^	0.98	2.44	3.308 (13)	148
C14—H14*B*⋯O26*A* ^i^	0.98	2.30	3.251 (9)	163
C14—H14*C*⋯O27*A* ^iii^	0.98	2.22	3.161 (8)	162
C15—H15*C*⋯O5	0.98	2.41	2.964 (4)	115
C19—H19⋯O26*A*	0.95	2.26	2.712 (9)	108

Symmetry codes: (i) 
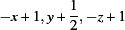
; (ii) 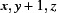; (iii) 
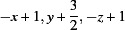
.

**Fig. 1.**
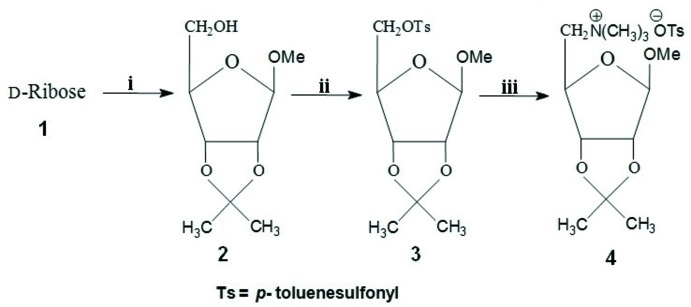
Preparation of N-[(methyl 5-deoxy-2,3-O-isopropylidene-β-D-ribofuranoside)-5-yl]-N,N,N-trimethylammonium tosylate, (4). Reagents: (i) SnCl2.2H2O, conc. H2SO4, acetone, MeOH; (ii) TsCl, py; (iii) 33% ethanolic solution of NMe3.

**Fig. 2.**
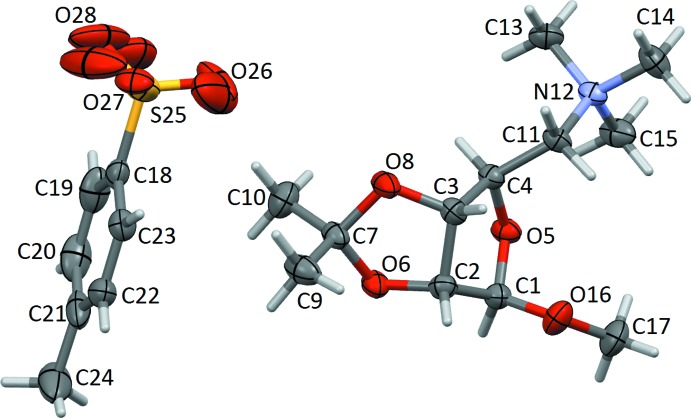
Molecular structure of N-[(methyl 5-deoxy-2,3-O-isopropylidene-β-D-ribofuranoside)-5-yl]-N,N,N-trimethylammonium tosylate, (4). Displacement ellipsoids drawn at 50% probability level. Oxygen atoms in the tosyl anion are disordered over two positions.

**Fig. 3.**
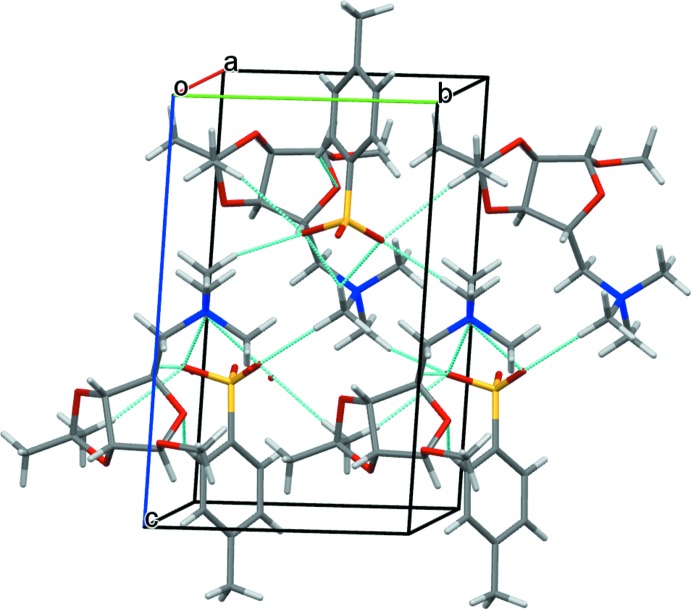
Packing diagram of 4, showing network of non-classical hydrogen bonds of the CH···O type and the hydrophilic / hydrophobic layers.

## supplementary crystallographic information

### Comment

Scientists the world over have been interested in quaternary ammonium compounds (QACs) for over a hundred years (Jones, 1984; Śliwa, 1996, Sajomsang *et al.*, 2009; Obłąk & Gamian, 2010). These salts display both inorganic properties (*e.g.* their excellent solubility in water) and organic ones, and their hydrophilic properties are due to their ionic nature. They are present in fabric softeners and corrosion inhibitors (Binks *et al.*, 2011; Singh *et al.*, 2009), they act as fungicides, pesticides and insecticides (Cruz-Guzman *et al.*, 2005), they exhibit antibacterial and antifungal activities and are therefore constituents of antimicrobial drugs (Rabea *et al.*, 2003; Belalia *et al.*, 2008; McDonnell & Russell,1999) and they are ingredients of shampoos and hair conditioners (Boethling, 1984; Levinson, 1999; Cross & Singer, 1994). Without doubt, QACs are used worldwide in industry, agriculture, healthcare and the home. Recent years have witnessed a resurgence of interest in the synthesis of QACs, especially their sugar derivatives, which have potential biological properties (Abel *et al.*, 2002; Blizzard *et al.*, 2002; Honda *et al.*, 1988; Thomas *et al.*, 2009; Maslov *et al.*, 2010).

One of our current research objectives is to find a correlation between the structure of the substituents around the quaternary nitrogen atom and the biological activity of the compounds concerned (Dmochowska *et al.*, 2011; Pellowska-Januszek *et al.*, 2004). The synthesis of QACs possessing a substituent that would increase the solubility of these salts appears to be interesting, and the incorporation into the QAC molecule of such a natural entity as a sugar unit could be very effective.

Synthetic work

Our research group synthesized numerous *N*-[(1,4-anhydro-5-deoxy-2,3-*O*-isopropylidene-D,L-ribitol)-5-yl]aminium tosylates (Dmochowska *et al.*, 2006; 2009; Skorupa *et al.*, 2004). We thought it would be very interesting to examine the synthesis of analogous QACs with methyl 2,3-*O*-isopropylidene-β-D-ribofuranoside and the influence of the *O*-Me substituent at the anomeric carbon atom on the course of the quaternization reaction at C-5 and on the conformation of the furanoic ring.

The synthesis of *N*-[(methyl 5-deoxy-2,3-*O*-isopropylidene-β-D-ribofuranoside)-5-yl]aminium tosylates began with commercially available D-ribose (1), Fig. 1, which was converted to methyl 2,3-*O*-isopropylidene-β-D-ribofuranoside (2), (Gosh & Liu 1996; Sairam *et al.*, 2003). Next, the hydroxyl group at C-5 of compound 2 was activated with *p*-toluenesulfonyl chloride using a well known method (Sarabia-Garcia & Lopez-Herrera, 1996). The idea was to investigate the reactions of methyl 2,3-*O*-isopropylidene-5-*O*-tosyl-β-D-ribofuranoside (3) with tertiary amines, *viz*. triethylamine, trimethylamine, 4-(*N,N*-dimethylamino)pyridine, isoquinoline, 2-methylpyridine and pyridine. All the newly synthesized *N*-[(methyl 5-deoxy-2,3-*O*-isopropylidene-β-D-ribofuranoside)-5-yl]aminium tosylates were water-soluble. Their structures were determined by NMR. Here we report the X-ray structure of the product with NMe_3_.

The reaction of 3 with 33% ethanolic solution of NMe_3_ (70° C, 48 h) yielded *N*-[(methyl 5-deoxy-2,3-*O*-isopropylidene-β-D-ribofuranoside)-5-yl]-*N,N,N*-trimethylammonium tosylate (4), (yield 67%). Its identity was also confirmed by ^1^H and ^13^C NMR spectra.

Description of the X-ray structure

Crystals belong to the monoclinic system, space group *P*2_1_. The asymmetric unit of (4) contains one tertiary ammonium cation and one tosyl anion and one and half molecules of solvate water (Fig. 2). The charged parts of both ions are directed towards the middle of the cell, forming a hydrophilic layer, perpendicular to the *c* axis (Fig. 3). The structure is reinforced by non-conventional C—H···O hydrogen bonds, which are formed perhaps due to the strong acceptor properties of anionic oxygen atoms (see Table 1). The region of disordered solvent is located in the hydrophilic layer and use of SQUEEZE recovered 16.8 e^-^ from a void of volume V = 101.8 Å^3^. Electron density, found in the void, was interpreted as coming from one and half molecules of water per one ionic pair. Naturally, this leads to formation of additional hydrogen bonds in the hydrophilic layer.

Ring puckering analysis (Cremer & Pople 1975; Spek, 2009) of 4 shows that the five-membered furan ring adopts the envelope C1 – *endo* conformation (with Q = 0.301 (3) Å, φ= 227.4 (6)°, P = 314.5 (3)° and τ_m_ (C2—C3) = 32.8 (2)°). Noteworthy, C1 atom is substituted by the methoxy group which may be the reason for increased stability of the conformation. In the analogous compound, substituted by hydrogen at position 1, not a carbon atom but an oxygen atom defined the envelope (see Table 2). Apparently, the opposite is true for compounds without the protection of OH groups. However, electrostatic forces also contribute significantly to the free energy balance, leading in the case of iodide to formation of the twisted furan ring.

The oxolane ring, O6—C2—C3—O8—C7, is best described as having the twisted conformation about the O6—C2 bond (with Q = 0.319 (3) Å, φ = 11.5 (6)°, P = 100.3 (3)° and τ_m_ (C3—O8) = 34.9 (2)°). To facilitate comparisons, we also included data on, published recently, *trans*-3,4 dihydroxy substituted analogue, (3,4-dihydroxyoxolan-2-yl)methyl 4-methylbenzenesulfonate (Dibrov *et al.*, 2010) whose conformation was not described by the authors (Q = 0.397 Å, φ = 245.4°, P = 333.7° and τ_m_ (C1—C8) = 41.7°).

### Experimental

General remarks

Commercial D-ribose (Fluka) was used. All reactions were monitored by thin-layer chromatography (TLC) on Kieselgel 60 F_254_ Silica Gel plates (E. Merck, 0.20 mm thickness) using eluent system (*v*/*v*) 3:1 CHCl_3_ – MeOH. The spots were detected by spraying with 5% ethanolic H_2_SO_4_ and charring. ^1^H and ^13^C NMR spectra (CDCl_3_, internal Me_4_Si) on a Varian Mercury 400 (400.49/100.70 MHz) instrument; positive-ion mode MALDITOF mass spectra on a Bruker Biflex III spectrometer with α-cyano-4-hydroxycinnamic acid as the matrix.

Synthesis of*N*-[(methyl 5-deoxy-2,3-*O*-isopropylidene-β-D-ribofuranoside)-5-yl]-*N,N,N*-trimethylammonium tosylate (4)

Methyl 2,3-*O*-isopropylidene-5-*O*-tosyl-β-D-ribofuranoside (3) (150 mg, 0.42 mmol) was dissolved in 33% ethanolic solution of NMe_3_ (0.31 ml). The solution was stored for 48 h in a screw-capped ampoule at 70°C, then the solvents were evaporated to dryness. The residue was dissolved in H_2_O, then extracted with CHCl_3_. The aqueous layer was evaporated and dried to yield quaternary aminium compound, which was recrystallized from 2-butanone, 4 (117.5 mg, 67%); M.pt: 348–351 K; [α]_D_^20^ -12.0 (*c* 1/5, H_2_O); ^1^H-NMR (D_2_O): δ 7.71–7.39 (2 d, each 2H, Ph), 5.22 (s, 1H, H-1), 4.89 (dd, 1H, H-3, *J*_2,3_ 5.6; *J*_3.4_ 1.4), 4.81 (d, 1H, H-2, *J*_2,3_ 6.0), 4.77 (m, 1H, H-4, *J*_4,5'_ 9.6), 3.69 (dd, 1H, H-5, *J*_4,5_ 2.6, *J*_5,5'_ 14.0), 3.55 (dd, 1H, H-5', *J*_4,5'_ 9.6), 3.49 (s, 3H, –OCH_3_), 3.26 (s, 9H, N(CH_3_)_3_), 2.42 (s, 3H, PhCH_3_), 1.56- 1.40 (2 s, each 3H, C(CH_3_)_2_); ^13^C NMR (H_2_O) δ 129.66-125.70 (C, Ph), 114.08 (C, *C*(CH_3_)_2_), 110.42 (C-1), 83.86 (C-2), 83.08 (C-3), 81.16 (C-4), 68.91 (C-5), 56.32 (OCH_3_), 54.26 (N(CH_3_)_3_), 25.62- 24.02 (C, C(*C*H_3_)_2_), 20.70 (C, Ph*C*H_3_); MALDI TOF– MS (CCA): m/*z* 246 ([*M*-OTs]^+^).

### Refinement

All hydrogen atoms were refined as riding with C–H distances in the range 0.95–1.00 Å and thermal ellipsoids *U*_iso_(H) = 1.5 *U*_iso_(C) for methyl groups or U_izo_(H) = 1.2 *U*_iso_(C) for aromatic or tertiary hydrogen atoms. Oxygen atoms of the tosyl group were refined as disordered over two positions with occupancies of 0.434 (12) and 0.566 (12). Bonds S—O in the tosyl group were constrained to be all equal. The structure was refined as a rotary twin with regard to rotation about the *c* axis with a small contribution of the second component of 0.0048 (6); R-indices with no twinning were wR^2^ = 0.143 and R_1_ = 0.052. Structure contains voids at *x,y,z* (0, 0, 1/2) filled with a disordered solvent, which is difficult to model. Use of program SQUEEZE (Spek, 2009) revealed electron density in that place equivalent to 16.8 e^-^, and volume of the void of *ca* 102 Å^3^. It can be attributed to the presence of one and half strongly disordered water molecule positioned in the hydrophilic layer. Three peaks in the asymmetric unit in the region could be found which may mean that position of water molecules can be coupled with the local disorder of the tosyl group oxygen atoms. The original reflection data were corrected for this electron density by the mentioned program. The formula, formula weight, F(000), density and absorption coefficient were corrected for water content in the CIF file. Conformational analysis parameters were calculated using *PLATON* program by Spek (2009).

### Crystal data

**Table:**

C_12_H_24_NO_4_^+^·C_7_H_7_O_3_S^−^·1.5H_2_O	*F*(000) = 478
*M**_r_* = 444.53	*D*_x_ = 1.292 Mg m^−^^3^
Monoclinic, *P*2_1_	Mo *K*α radiation, λ = 0.71073 Å
Hall symbol: P 2yb	Cell parameters from 3067 reflections
*a* = 11.4896 (15) Å	θ = 2–30°
*b* = 7.9311 (11) Å	µ = 0.19 mm^−^^1^
*c* = 13.4853 (17) Å	*T* = 200 K
β = 111.619 (12)°	Block, colourless
*V* = 1142.4 (3) Å^3^	0.37 × 0.2 × 0.17 mm
*Z* = 2	

### Data collection

**Table:**

Kuma KM4CCD (Sapphire2 detector) diffractometer	*R*_int_ = 0.038
Graphite monochromator	θ_max_ = 26°, θ_min_ = 2.9°
ω scans, 1 deg frames	*h* = −14→14
26053 measured reflections	*k* = −9→9
4481 independent reflections	*l* = −16→16
4325 reflections with *I* > 2σ(*I*)	

### Refinement

**Table:**

Refinement on *F*^2^	Secondary atom site location: difference Fourier map
Least-squares matrix: full	Hydrogen site location: inferred from neighbouring sites
*R*[*F*^2^ > 2σ(*F*^2^)] = 0.051	H-atom parameters constrained
*wR*(*F*^2^) = 0.139	*w* = 1/[σ^2^(*F*_o_^2^) + (0.0737*P*)^2^ + 0.6709*P*] where *P* = (*F*_o_^2^ + 2*F*_c_^2^)/3
*S* = 1.07	(Δ/σ)_max_ = 0.007
4481 reflections	Δρ_max_ = 0.40 e Å^−^^3^
283 parameters	Δρ_min_ = −0.31 e Å^−^^3^
40 restraints	Absolute structure: Flack & Bernardinelli (1999)
Primary atom site location: structure-invariant direct methods	Flack parameter: 0.08 (12)

### Special details

**Table:**

Geometry. All s.u.'s (except the s.u. in the dihedral angle between two l.s. planes) are estimated using the full covariance matrix. The cell s.u.'s are taken into account individually in the estimation of s.u.'s in distances, angles and torsion angles; correlations between s.u.'s in cell parameters are only used when they are defined by crystal symmetry. An approximate (isotropic) treatment of cell s.u.'s is used for estimating s.u.'s involving l.s. planes.
Refinement. Refinement of *F*^2^ against ALL reflections. The weighted *R*-factor *wR* and goodness of fit *S* are based on *F*^2^, conventional *R*-factors *R* are based on *F*, with *F* set to zero for negative *F*^2^. The threshold expression of *F*^2^ > 2σ(*F*^2^) is used only for calculating *R*-factors(gt) *etc*. and is not relevant to the choice of reflections for refinement. *R*-factors based on *F*^2^ are statistically about twice as large as those based on *F*, and *R*- factors based on ALL data will be even larger.

### Fractional atomic coordinates and isotropic or equivalent isotropic displacement parameters (Å^2^)

**Table:**

	*x*	*y*	*z*	*U*_iso_*/*U*_eq_	Occ. (<1)
C1	0.4006 (3)	0.9674 (4)	0.8428 (2)	0.0299 (6)	
H1	0.4517	1.0147	0.9144	0.036*	
C2	0.4217 (3)	0.7801 (4)	0.8398 (2)	0.0294 (6)	
H2	0.3676	0.7123	0.8683	0.035*	
C3	0.3957 (3)	0.7445 (4)	0.7215 (2)	0.0284 (6)	
H3	0.3089	0.701	0.6836	0.034*	
C4	0.4153 (3)	0.9195 (3)	0.6776 (2)	0.0261 (6)	
H4	0.4913	0.9144	0.6584	0.031*	
O5	0.43649 (19)	1.0397 (3)	0.76273 (15)	0.0295 (5)	
O6	0.5509 (2)	0.7393 (3)	0.88856 (15)	0.0322 (5)	
C7	0.5743 (3)	0.6006 (4)	0.8296 (2)	0.0314 (6)	
O8	0.4872 (2)	0.6252 (3)	0.72143 (15)	0.0392 (5)	
C9	0.5476 (4)	0.4340 (4)	0.8700 (3)	0.0447 (8)	
H9A	0.5643	0.3425	0.8281	0.067*	
H9B	0.4597	0.43	0.8628	0.067*	
H9C	0.6015	0.4207	0.9452	0.067*	
C10	0.7061 (3)	0.6147 (5)	0.8315 (3)	0.0457 (8)	
H10A	0.7229	0.5206	0.7916	0.069*	
H10B	0.7656	0.6114	0.9054	0.069*	
H10C	0.7154	0.7215	0.7985	0.069*	
C11	0.3025 (3)	0.9654 (4)	0.5787 (2)	0.0282 (6)	
H11A	0.2815	0.8671	0.53	0.034*	
H11B	0.2304	0.9857	0.6005	0.034*	
N12	0.3178 (2)	1.1180 (3)	0.51680 (16)	0.0272 (5)	
C13	0.4218 (3)	1.0949 (4)	0.4768 (2)	0.0378 (7)	
H13A	0.4081	0.9909	0.4347	0.057*	
H13B	0.5016	1.0875	0.5374	0.057*	
H13C	0.4239	1.1911	0.4319	0.057*	
C14	0.1974 (3)	1.1358 (5)	0.4218 (2)	0.0424 (7)	
H14A	0.1277	1.1511	0.4463	0.064*	
H14B	0.1833	1.034	0.3777	0.064*	
H14C	0.2028	1.2339	0.3796	0.064*	
C15	0.3408 (4)	1.2769 (4)	0.5807 (3)	0.0407 (8)	
H15A	0.2732	1.2939	0.6076	0.061*	
H15B	0.3434	1.3725	0.5354	0.061*	
H15C	0.4209	1.2686	0.6409	0.061*	
O16	0.2726 (2)	0.9863 (3)	0.8220 (2)	0.0411 (6)	
C17	0.2344 (4)	1.1544 (6)	0.8338 (3)	0.0527 (9)	
H17A	0.144	1.1564	0.8176	0.079*	
H17B	0.2545	1.2296	0.7847	0.079*	
H17C	0.2787	1.1924	0.9073	0.079*	
C18	0.8291 (3)	0.1549 (4)	0.8487 (2)	0.0322 (6)	
C19	0.9296 (3)	0.2527 (5)	0.9119 (4)	0.0503 (9)	
H19	0.9773	0.3154	0.8802	0.06*	
C20	0.9596 (3)	0.2581 (5)	1.0210 (4)	0.0550 (10)	
H20	1.028	0.3257	1.0638	0.066*	
C21	0.8916 (3)	0.1665 (5)	1.0694 (3)	0.0497 (10)	
C22	0.7906 (3)	0.0723 (4)	1.0053 (3)	0.0391 (7)	
H22	0.7424	0.0101	1.0368	0.047*	
C23	0.7587 (3)	0.0678 (4)	0.8952 (2)	0.0313 (6)	
H23	0.6881	0.0044	0.8519	0.038*	
C24	0.9289 (5)	0.1691 (8)	1.1889 (3)	0.086 (2)	
H24A	1.0139	0.124	1.2227	0.13*	
H24B	0.8705	0.0995	1.2089	0.13*	
H24C	0.9265	0.2852	1.2129	0.13*	
S25	0.78821 (10)	0.14712 (13)	0.70869 (7)	0.0533 (3)	
O26	0.6888 (12)	0.2652 (11)	0.6587 (9)	0.063 (4)	0.434 (12)
O27	0.7138 (7)	−0.0238 (7)	0.6715 (4)	0.039 (2)	0.434 (12)
O28	0.8913 (7)	0.138 (2)	0.6800 (6)	0.093 (6)	0.434 (12)
O26A	0.8903 (7)	0.2787 (11)	0.7015 (6)	0.074 (2)	0.566 (12)
O27A	0.8177 (15)	−0.0076 (8)	0.6870 (7)	0.115 (6)	0.566 (12)
O28A	0.6753 (10)	0.235 (2)	0.6701 (10)	0.109 (6)	0.566 (12)

### Atomic displacement parameters (Å^2^)

**Table:**

	*U*^11^	*U*^22^	*U*^33^	*U*^12^	*U*^13^	*U*^23^
C1	0.0336 (15)	0.0316 (15)	0.0258 (13)	0.0032 (12)	0.0123 (11)	0.0008 (11)
C2	0.0327 (15)	0.0305 (15)	0.0276 (13)	−0.0006 (12)	0.0142 (11)	0.0016 (11)
C3	0.0305 (15)	0.0277 (14)	0.0249 (13)	−0.0006 (12)	0.0077 (11)	0.0011 (11)
C4	0.0288 (14)	0.0267 (14)	0.0220 (12)	−0.0006 (11)	0.0085 (10)	−0.0003 (10)
O5	0.0378 (11)	0.0260 (10)	0.0245 (9)	−0.0026 (8)	0.0112 (8)	−0.0017 (8)
O6	0.0363 (11)	0.0317 (11)	0.0238 (9)	0.0047 (9)	0.0053 (8)	−0.0036 (8)
C7	0.0411 (16)	0.0258 (15)	0.0239 (13)	0.0045 (12)	0.0080 (11)	−0.0005 (11)
O8	0.0576 (14)	0.0338 (12)	0.0235 (9)	0.0153 (11)	0.0117 (9)	0.0003 (9)
C9	0.071 (2)	0.0285 (17)	0.0331 (16)	0.0007 (16)	0.0170 (16)	0.0024 (13)
C10	0.0428 (18)	0.042 (2)	0.0556 (19)	0.0086 (16)	0.0216 (15)	−0.0026 (16)
C11	0.0317 (15)	0.0285 (15)	0.0232 (12)	−0.0037 (12)	0.0089 (11)	0.0035 (11)
N12	0.0320 (12)	0.0271 (12)	0.0213 (10)	−0.0037 (10)	0.0083 (9)	−0.0014 (9)
C13	0.0438 (17)	0.0425 (19)	0.0320 (14)	−0.0032 (15)	0.0199 (13)	0.0026 (13)
C14	0.0395 (17)	0.0455 (19)	0.0325 (14)	0.0001 (15)	0.0019 (12)	0.0118 (15)
C15	0.064 (2)	0.0266 (16)	0.0340 (16)	−0.0035 (15)	0.0214 (15)	−0.0020 (13)
O16	0.0346 (12)	0.0409 (13)	0.0544 (13)	0.0037 (10)	0.0242 (10)	−0.0032 (11)
C17	0.055 (2)	0.051 (2)	0.058 (2)	0.0229 (19)	0.0277 (18)	0.0015 (19)
C18	0.0295 (14)	0.0293 (15)	0.0397 (15)	0.0087 (13)	0.0150 (12)	0.0041 (13)
C19	0.0292 (17)	0.0359 (18)	0.085 (3)	−0.0051 (14)	0.0198 (17)	0.0035 (18)
C20	0.0311 (18)	0.047 (2)	0.071 (3)	−0.0012 (16)	0.0001 (17)	−0.020 (2)
C21	0.0426 (19)	0.052 (2)	0.0374 (17)	0.0237 (17)	−0.0054 (14)	−0.0122 (16)
C22	0.0429 (18)	0.0407 (18)	0.0365 (16)	0.0116 (14)	0.0178 (14)	0.0057 (13)
C23	0.0291 (14)	0.0267 (14)	0.0343 (15)	−0.0008 (11)	0.0073 (12)	−0.0011 (11)
C24	0.086 (3)	0.113 (4)	0.037 (2)	0.054 (3)	−0.005 (2)	−0.022 (2)
S25	0.0711 (7)	0.0562 (6)	0.0434 (4)	0.0187 (5)	0.0335 (4)	0.0176 (4)
O26	0.110 (10)	0.025 (3)	0.026 (4)	0.011 (4)	−0.006 (4)	−0.004 (3)
O27	0.055 (4)	0.034 (3)	0.028 (3)	−0.011 (3)	0.017 (3)	−0.012 (2)
O28	0.060 (5)	0.185 (16)	0.046 (4)	−0.043 (7)	0.034 (3)	−0.015 (6)
O26A	0.087 (5)	0.082 (5)	0.076 (4)	−0.001 (4)	0.057 (4)	0.013 (4)
O27A	0.261 (18)	0.043 (3)	0.077 (5)	0.007 (6)	0.105 (8)	−0.005 (3)
O28A	0.050 (5)	0.211 (15)	0.057 (5)	0.017 (6)	0.008 (4)	0.027 (6)

### Geometric parameters (Å, º)

**Table:**

C1—O16	1.400 (4)	C14—H14A	0.98
C1—O5	1.411 (3)	C14—H14B	0.98
C1—C2	1.508 (4)	C14—H14C	0.98
C1—H1	1	C15—H15A	0.98
C2—O6	1.421 (4)	C15—H15B	0.98
C2—C3	1.537 (4)	C15—H15C	0.98
C2—H2	1	O16—C17	1.431 (5)
C3—O8	1.414 (4)	C17—H17A	0.98
C3—C4	1.557 (4)	C17—H17B	0.98
C3—H3	1	C17—H17C	0.98
C4—O5	1.442 (3)	C18—C23	1.377 (4)
C4—C11	1.522 (4)	C18—C19	1.391 (5)
C4—H4	1	C18—S25	1.771 (3)
O6—C7	1.439 (3)	C19—C20	1.383 (6)
C7—O8	1.447 (3)	C19—H19	0.95
C7—C9	1.503 (4)	C20—C21	1.393 (6)
C7—C10	1.510 (5)	C20—H20	0.95
C9—H9A	0.98	C21—C22	1.383 (5)
C9—H9B	0.98	C21—C24	1.508 (5)
C9—H9C	0.98	C22—C23	1.393 (4)
C10—H10A	0.98	C22—H22	0.95
C10—H10B	0.98	C23—H23	0.95
C10—H10C	0.98	C24—H24A	0.98
C11—N12	1.516 (3)	C24—H24B	0.98
C11—H11A	0.99	C24—H24C	0.98
C11—H11B	0.99	S25—O27A	1.334 (6)
N12—C13	1.493 (4)	S25—O28	1.377 (7)
N12—C15	1.494 (4)	S25—O28A	1.396 (9)
N12—C14	1.507 (4)	S25—O26	1.438 (9)
C13—H13A	0.98	S25—O27	1.582 (6)
C13—H13B	0.98	S25—O26A	1.599 (7)
C13—H13C	0.98		
			
O16—C1—O5	112.6 (2)	N12—C14—H14A	109.5
O16—C1—C2	105.5 (3)	N12—C14—H14B	109.5
O5—C1—C2	106.5 (2)	H14A—C14—H14B	109.5
O16—C1—H1	110.7	N12—C14—H14C	109.5
O5—C1—H1	110.7	H14A—C14—H14C	109.5
C2—C1—H1	110.7	H14B—C14—H14C	109.5
O6—C2—C1	111.3 (2)	N12—C15—H15A	109.5
O6—C2—C3	102.1 (2)	N12—C15—H15B	109.5
C1—C2—C3	103.7 (2)	H15A—C15—H15B	109.5
O6—C2—H2	113	N12—C15—H15C	109.5
C1—C2—H2	113	H15A—C15—H15C	109.5
C3—C2—H2	113	H15B—C15—H15C	109.5
O8—C3—C2	105.2 (2)	C1—O16—C17	114.8 (3)
O8—C3—C4	112.7 (2)	O16—C17—H17A	109.5
C2—C3—C4	103.3 (2)	O16—C17—H17B	109.5
O8—C3—H3	111.7	H17A—C17—H17B	109.5
C2—C3—H3	111.7	O16—C17—H17C	109.5
C4—C3—H3	111.7	H17A—C17—H17C	109.5
O5—C4—C11	112.3 (2)	H17B—C17—H17C	109.5
O5—C4—C3	107.0 (2)	C23—C18—C19	119.6 (3)
C11—C4—C3	110.6 (2)	C23—C18—S25	119.9 (2)
O5—C4—H4	109	C19—C18—S25	120.4 (3)
C11—C4—H4	109	C20—C19—C18	119.7 (3)
C3—C4—H4	109	C20—C19—H19	120.2
C1—O5—C4	109.2 (2)	C18—C19—H19	120.2
C2—O6—C7	107.1 (2)	C19—C20—C21	121.2 (3)
O6—C7—O8	104.7 (2)	C19—C20—H20	119.4
O6—C7—C9	111.6 (2)	C21—C20—H20	119.4
O8—C7—C9	109.0 (3)	C22—C21—C20	118.4 (3)
O6—C7—C10	109.0 (3)	C22—C21—C24	121.1 (5)
O8—C7—C10	108.9 (3)	C20—C21—C24	120.4 (4)
C9—C7—C10	113.4 (3)	C21—C22—C23	120.7 (3)
C3—O8—C7	109.1 (2)	C21—C22—H22	119.7
C7—C9—H9A	109.5	C23—C22—H22	119.7
C7—C9—H9B	109.5	C18—C23—C22	120.3 (3)
H9A—C9—H9B	109.5	C18—C23—H23	119.9
C7—C9—H9C	109.5	C22—C23—H23	119.9
H9A—C9—H9C	109.5	C21—C24—H24A	109.5
H9B—C9—H9C	109.5	C21—C24—H24B	109.5
C7—C10—H10A	109.5	H24A—C24—H24B	109.5
C7—C10—H10B	109.5	C21—C24—H24C	109.5
H10A—C10—H10B	109.5	H24A—C24—H24C	109.5
C7—C10—H10C	109.5	H24B—C24—H24C	109.5
H10A—C10—H10C	109.5	O27A—S25—O28	65.0 (8)
H10B—C10—H10C	109.5	O27A—S25—O28A	131.0 (9)
N12—C11—C4	116.1 (2)	O28—S25—O28A	134.3 (8)
N12—C11—H11A	108.3	O27A—S25—O26	135.9 (7)
C4—C11—H11A	108.3	O28—S25—O26	121.2 (8)
N12—C11—H11B	108.3	O27A—S25—O27	45.1 (6)
C4—C11—H11B	108.3	O28—S25—O27	106.7 (7)
H11A—C11—H11B	107.4	O28A—S25—O27	89.6 (7)
C13—N12—C15	108.5 (2)	O26—S25—O27	99.6 (5)
C13—N12—C14	108.1 (2)	O27A—S25—O26A	109.6 (6)
C15—N12—C14	108.8 (3)	O28A—S25—O26A	104.6 (7)
C13—N12—C11	111.8 (2)	O26—S25—O26A	91.2 (7)
C15—N12—C11	112.8 (2)	O27—S25—O26A	149.6 (3)
C14—N12—C11	106.7 (2)	O27A—S25—C18	106.1 (4)
N12—C13—H13A	109.5	O28—S25—C18	112.6 (3)
N12—C13—H13B	109.5	O28A—S25—C18	103.0 (6)
H13A—C13—H13B	109.5	O26—S25—C18	109.3 (5)
N12—C13—H13C	109.5	O27—S25—C18	105.6 (2)
H13A—C13—H13C	109.5	O26A—S25—C18	97.4 (3)
H13B—C13—H13C	109.5		
			
O16—C1—C2—O6	−163.4 (2)	C9—C7—O8—C3	105.0 (3)
O5—C1—C2—O6	76.7 (3)	C10—C7—O8—C3	−130.9 (3)
O16—C1—C2—C3	87.6 (3)	O5—C4—C11—N12	70.6 (3)
O5—C1—C2—C3	−32.4 (3)	C3—C4—C11—N12	−170.0 (2)
O6—C2—C3—O8	24.8 (3)	C4—C11—N12—C13	60.4 (3)
C1—C2—C3—O8	140.5 (2)	C4—C11—N12—C15	−62.1 (3)
O6—C2—C3—C4	−93.5 (3)	C4—C11—N12—C14	178.4 (3)
C1—C2—C3—C4	22.2 (3)	O5—C1—O16—C17	−71.4 (3)
O8—C3—C4—O5	−118.6 (2)	C2—C1—O16—C17	172.8 (3)
C2—C3—C4—O5	−5.5 (3)	C23—C18—C19—C20	1.6 (5)
O8—C3—C4—C11	118.8 (3)	S25—C18—C19—C20	179.5 (3)
C2—C3—C4—C11	−128.1 (2)	C18—C19—C20—C21	0.4 (6)
O16—C1—O5—C4	−85.3 (3)	C19—C20—C21—C22	−1.6 (6)
C2—C1—O5—C4	30.0 (3)	C19—C20—C21—C24	177.7 (4)
C11—C4—O5—C1	106.6 (3)	C20—C21—C22—C23	0.8 (5)
C3—C4—O5—C1	−14.9 (3)	C24—C21—C22—C23	−178.5 (3)
C1—C2—O6—C7	−144.6 (2)	C19—C18—C23—C22	−2.4 (5)
C3—C2—O6—C7	−34.5 (3)	S25—C18—C23—C22	179.6 (2)
C2—O6—C7—O8	31.5 (3)	C21—C22—C23—C18	1.2 (5)
C2—O6—C7—C9	−86.3 (3)	C23—C18—S25—O27	−24.9 (4)
C2—O6—C7—C10	147.8 (3)	C19—C18—S25—O27	157.2 (4)
C2—C3—O8—C7	−6.3 (3)	C23—C18—S25—O26A	175.2 (4)
C4—C3—O8—C7	105.6 (3)	C19—C18—S25—O26A	−2.7 (4)
O6—C7—O8—C3	−14.5 (3)		

### Hydrogen-bond geometry (Å, º)

**Table:**

*D*—H···*A*	*D*—H	H···*A*	*D*···*A*	*D*—H···*A*
C13—H13*A*···O28*A*^i^	0.98	2.45	3.412 (15)	167
C13—H13*B*···O28*A*^ii^	0.98	2.44	3.308 (13)	148
C14—H14*B*···O26*A*^i^	0.98	2.30	3.251 (9)	163
C14—H14*C*···O27*A*^iii^	0.98	2.22	3.161 (8)	162
C15—H15*C*···O5	0.98	2.41	2.964 (4)	115
C19—H19···O26*A*	0.95	2.26	2.712 (9)	108

Symmetry codes: (i) −*x*+1, *y*+1/2, −*z*+1; (ii) *x*, *y*+1, *z*; (iii) −*x*+1, *y*+3/2, −*z*+1.

### Conformations of six selected compounds. Atom numbering schema taken from the original papers. Qualitative puckering descriptors (envelope, twisted) are the ones closest to the observed data.

**Table:**

Conformation of the furanoic ring	Conformation of the dioxolane ring	C1 substitution	C2–C3 substitution	C4 substitution	Reference
Envelope on the O5 atom (E_0_)	Envelope on C7 (E^1^)	H	2,3-*O*-isopropylidene	CH_2_NMe_3_(+), OTs(-)	Dmochowska *et al.* (2006); Skorupa *et al.* (2004)
Twisted about the O5–C1 bond (^0^T_1_)	Envelope on C7 (E_0_)	H	2,3-*O*-isopropylidene	CH_2_NMe_3_(+), I(-)	Dmochowska *et al.* (2006)
Envelope on the C1 atom (E^1^)	Twisted about the O6-C2 bond(^0^T_1_)	OCH_3_	2,3-*O*-isopropylidene	CH_2_NMe_3_(+), OTs(-)	this work
Envelope on the O5 atom (E^0^)	Twisted about the O18-C20 bond (^2^T_3_)	H	2,3-*O*-isopropylidene	CH_2_OTs	Dmochowska *et al.* (2009)
Envelope on the C3 atom (E^3^)		H	*cis*-2,3-dihydroxy	CH_2_OTs	Dmochowska *et al.* (2009)
Envelope on the C1 atom (E^2^)		H	*trans*-2,3-dihydroxy	CH_2_OTs	Dibrov *et al.* (2010)
